# Proceedings from the V Brazilian Meeting on Research Integrity, Science and Publication Ethics (V BRISPE): Porto Alegre, Brazil. 26-28 October 2018

**DOI:** 10.1186/s41073-020-0090-6

**Published:** 2020-03-03

## 1 Proceedings from the V Brazilian Meeting on Research Integrity, Science and Publication Ethics (V BRISPE)

### Carla Bonan^1^ (Chair), Claude Pirmez^2^ (Co-Chair), Denise Machado^1^, Edson Watanabe^3^, Martha Sorenson^4^, Sonia Vasconcelos^4,5^ (Proceedings Organizers)

#### ^1^Pontifical Catholic University of Rio Grande do Sul (PUCRS); ^2^Oswaldo Cruz Foundation (FIOCRUZ); ^3^Electrical Engineering Program (PEE), Institute Alberto Luiz Coimbra of Graduate Studies and Research in Engineering (COPPE), Federal University of Rio de Janeiro (UFRJ), Rio de Janeiro, Brazil; ^4^Science Education Program (PEGeD), Institute of Medical Biochemistry Leopoldo de Meis (IBqM)/UFRJ; ^5^Professional Masters Program (MP- EGeD)/IBqM/UFRJ, Rio de Janeiro, Brazil

*The **Brazilian Meeting on Research Integrity, Science and Publication Ethics** (**BRISPE**) is the major forum discussing these topics in Brazil.

In 2010, the **I BRISPE** (www.ibrispe.coppe.ufrj.br) focused on research integrity issues related to research projects, to the submission and review process of manuscripts, and to authorship.

In 2012, the **II BRISPE** (www.iibrispe.coppe.ufrj.br) explored research integrity and leadership in science, considering the Brazilian scientific leadership in Latin America. The II BRISPE led to the Joint Statement on Research Integrity, (www.iibrispe.coppe.ufrj.br/images/IIBRISPE/JoinStatement/JointStatementonResearchIntegrity_IIBRISPE_2012_English.pdf).

In 2014, the **III BRISPE** (http://www.fapesp.br/8788) focused on institutional policies to foster research integrity initiatives at universities and research centers in Brazil. For Brazilian academia, the III BRISPE was held as a preparatory meeting for the 4^th^ World Conference on Research Integrity, which was held in Rio de Janeiro, in 2015 (www.wcri2015.org). In 2016, the **IV BRISPE** (http://eventus.com.br/brispe2016/) addressed the role of mentors, editors and funders to strengthen a research integrity culture in Brazilian science.

In 2018, the **V BRISPE** (http://www.pucrs.br/eventos/inst/vbrispe-2/) focused on research integrity and the reliability of the research record, exploring the role of graduate programs. The event was held at the Pontifical University of Rio Grande Sul (PUCRS). For this fifth edition of the meeting, there was a call for submissions for poster session on research and education and on science policy. From 44 submissions, 34 were accepted for posters and 24 for publication in the Proceedings.

The **Proceedings of the V BRISPE** contain the abstracts of poster presentations of authors who agreed to have them published.

The **VI BRISPE** will be held in December, 2020.

**Acknowledgments**

We would like to thank all members of the organizing and program committees (http://www.pucrs.br/eventos/inst/vbrispe-2/), who had a crucial role in the organization of the V BRISPE. Dr. Martha Sorenson is also acknowledged for her important assistance with the organization of these Proceedings.

*This is a general introductory text to BRISPE editions, describing the focus of the meeting for each one. A similar description was made in the Proceedings for the IV BRISPE, https://researchintegrityjournal.biomedcentral.com/articles/10.1186/s41073-017-0035-x

## 2 Music and music education research in Brazil: Ethical issues

### Cristina Rolim Wolffenbüttel

#### Rio Grande do Sul State University, Montenegro, Rio Grande do Sul, Brazil

Research in music and music education have expanded in Brazil. This growth can be observed in publications of the Brazilian Association of Musical Education (ABEM) journals [1] and Opus journals [2]. Based on this progress, one question raised is How does ethics present itself in these publications? In order to investigate the ethical aspects of the articles published in both the ABEM and Opus journals, this research collected data over 29 years from a corpus composed of 80 pieces, using the following terms: ethics, research ethics and ethics committee. Our results show that despite advances in ethics regulation for human-subject research in Brazil, particularly for studies in Social Sciences the Humanities (SSH), and the need for research in music and music education to consider such regulation, the methodological procedures do not mention ethical review of protocols. Resolution No. 510/2016 [3] for example, establishes the norms applicable to research in SSH, and one of the points is to clarify possible risks for participants taking part in different types of study. We found 19 articles, 11 in the Opus journals and eight in the ABEM journals, and only 10 articles, four and six articles in the Opus and ABEM journals, respectively. The reference to research ethics and integrity is diverse, appearing from the care in the data collection throughout the research to relations between ethics and aesthetics. Although these ethics mentions are not part of the research tradition in music and music education, we believe ethical concerns described in projects in these fields should increase with the advances in ethics regulation in the country.

**References**

1. ABEM [Brazilian Association for Music Education]. Quem somos. Available in: http://abemeducacaomusical.com.br/abem.asp#t1. [Access in: 2018 May 13].

2. OPUS.[e-Journal of the National Association for Research and Graduate Studies in Music] Sobre a Opus. Available at: https://www.anppom.com.br/revista/index.php/opus/index. [Access in: 2018 May 13]

3. BRASIL. Ministério da Saúde. Conselho Nacional de Saúde. Resolução n.° 510, de 7 de abril de 2016. Diário Oficial [da] República Federativa do Brasil, Brasília, DF, 24 maio 2016. Seção 1. p. 44-46. Available at: http://conselho.saude.gov.br/resolucoes/2016/Reso510.pdf. [Access in: 2018 May 13]

**Keywords:** Music, music education, research, ethics.

## 3 Research integrity in scientific guidelines to authors submitting manuscripts to Qualis periodicals A1 and A2 in the Education area

### Luís P Mercado, Janyelle M Bento, Leonardo F T da Silva

#### Graduate Program in Education, Federal University of Alagoas, AL, Brazil

##### **Correspondence:** Luís P Mercado

The paper presents an analysis of research integrity policies [1,2,3,4,5,6] reflected on publication standards contained in the instructions to the authors of the scientific journals rated A1 and A2 in the area of education. This is a qualitative study exploring authors’ guidelines for submission of manuscripts to journals classified as Qualis A1 and A2 in the education area, in the 2013-2016 quadrennial evaluation of the Coordination for the Improvement of Higher Education Personnel (CAPES). In this survey, the following categories were analyzed: (1) Procedures for the recording and publishing retractions and expressions of concern; (2) For disclosure of conflicts of interest and attribution of authorship on papers; (3) Requirements forauthorship in unpublished material; (4) Guidelines for data acquisition or analysis and interpretation of data from other publications; (5) Evaluation of the textual content of the articles and procedures taken when identifying plagiarisms, duplicate submissions, manuscripts already published and possible fraud in research; (6) Guidelines for peer review; and (7) Cancellation of an article. The analysis of the data was based on the content of guidelines to the authors in the pages of the journals researched from the categories and descriptors provided in the objectives. We found that there is a long way to go for the dissemination and consolidation of integrity rules in research, since less than 1/3 of the 500 journals collected has attention to this theme in their guidelines to authors. Some journals still prefer the use of plagiarism detectors, in this case detecting plagiarism as a research fraud, rather than trying to establish a “culture” of promoting research integrity, with guidelines and standards for performing good research practices. The results allowed us to assess research integrity policies for journals A1 and A2 in the education area, offering a brief panorama of the way they have been presented in this sample of journals, which shows that research integrity policies are in their infancy in these journals.

**References**

1. Fernandes, MR, Queiroz MC, de Moraes MR, Barbosa MA, Sousa AL. Padrões éticos adotados pelas revistas científicas brasileiras das especialidades médicas. **Rev Assoc Méd Bras**, 57 (3): 267-271, Available at: http://www.scielo.br/scielo.php?script=sci_arttext&pid=S0104-42302011000300007&lng=en&nrm=iso&tlng=pt&ORIGINALLANG=pt. [Access 2016 Jul 10].

2. Resnik DB, Patrone D, Peddada S. Research misconduct polices of social science journals and impact factor. Account Res**:** Policies and Quality Assurance**,**2010; 17(2):79-84.

3. Steneck, N. Fostering integrity in research: definitions, current knowledge, and future directions. Sci Eng Ethics, 2006; (12):53-74, 2006.

4. Thomaz PG, Assad RS, Moreira LF. Uso do fator de impacto e do índice H para avaliar pesquisadores e publicações. Arq Bras Cardiol 2011; [Accessed 2015 May 2]; 96(2):90-93. Available at: http://www.scielo.br/pdf/abc/v96n2/v96n2a01.pdf

5. Vasconcelos SM. Integridade científica e correção da literatura: Desafios na comunicação científica. In: Dallari SG et al., editors. Seminários: A Ética e a Universidade 2012-2013.São Paulo: Comissão de Ética da USP; 2014. p.1-11.

6. Watanabe EH. A não linearidade entre a reação de quem copia e de quem é copiado. EstudAv.2014 Apr; 28(80):199-212.

**Keywords:** Scientific Integrity; Ethics in Research; Scientific Publishing; Scientific Publishing.

## 4 Retractions for plagiarism: Would they reflect the extent of the problem in the communication of science?

### Mariana D Ribeiro, Sonia M R Vasconcelos

#### Science Education Program, Institute of Medical Biochemistry Leopoldo de Meis (IBqM), Federal University of Rio de Janeiro (UFRJ), Rio de Janeiro, RJ, Brazil

##### **Correspondence:** Mariana D Ribeiro

In a retrospective study (2000-2015) focusing on BiomedCentral publications, Moylan & Kowalczuk [1] showed that 0.07% of the total of articles published were retracted, with plagiarism accounting for 16%. This percentage is lower than that reported for publications retracted between 2008-2014 in the major Latin American and Caribbean scientific database [2] but higher than those reported in other studies [3,4] Among methodological issues influencing these different results, we may add that a given plagiarism case would be handled differently by different journals – some may issue a correction and others may issue a retraction. Some others may handle plagiarism without using a formal editorial resource [5]. Looking at retraction notices in Retraction Watch (RW) from 2013-2015, we collected 1623 retractions. We identified the country of origin and fields, according to the Journal of Citation Reports [6]. Among these 1,623 retractions, 240 were for plagiarism, distributed among 44 countries. We found prevalence for India, Iran and Italy for that period. For a broader panorama of the problem, we have now looked at retractions for plagiarism from the Retraction Database, a comprehensive dataset recently designed. We collected 986 retractions for 2015 and found that 16% were attributed to plagiarism, classified by the country of the corresponding author. This work is underway, and our approach considers the heterogeneous panorama that the literature has presented. Also, we look at countries with different research traditions, considering linguistic and cultural factors that so far have received limited attention.

**References**

1. Moylan E, Kowalczuk M. Why articles are retracted: A retrospective cross-sectional study of retraction notices at BioMed Central. BMJ Open. 2016;6(11):e012047.

2. Almeida R, de Albuquerque Rocha K, Catelani F, Fontes-Pereira A, Vasconcelos S. Plagiarism allegations account for most retractions in major Latin American/Caribbean databases. Sci Eng Ethics. 2015;22(5):1447-1456.

3. Steen R, Casadevall A, Fang F. Why has the number of scientific retractions increased?. PLoS ONE. 2013;8(7):e68397.

4. Grieneisen M, Zhang M. A comprehensive survey of retracted articles from the scholarly literature. PLoS ONE. 2012;7(10):e44118.

5. Wager E, Barbour V, Yentis S, Kleinert S. Retractions: Guidance from the Committee on Publication Ethics (COPE). Croat Med J. 2009;50(6):532-535.

6. Ribeiro M, Vasconcelos S. Retractions covered by Retraction Watch in the 2013– 2015 period: prevalence for the most productive countries. Scientometrics. 2018;114(2):719-734.

**Keywords:** Retractions, plagiarism, science communication.

## 5 Promotion of research integrity in a *lato sensu* specialization course at Adolfo Lutz Institute, São Paulo, SP, 2018, using video lessons and active teaching methodologies

### Bráulio C Machado^1^, Luz M Trujillo^2^, Raquel A Fazioli^3^, Adriana P Vicentini^1^, Adriana AB Almodóvar^1^, Regina M Catarino^1^

#### ^1^Research Integrity Committee, Adolfo Lutz Institute, São Paulo, SP, Brazil; ^2^Research Ethics Committee for the Protection of Human Subjects, Adolfo Lutz Institute, São Paulo, SP, Brazil; ^3^Committee for Ethical Use of Animals in Research, Adolfo Lutz Institute, São Paulo, SP, Brazil

##### **Correspondence:** Bráulio C Machado

The Research Integrity Committee (CIP) was officially implemented in Adolfo Lutz Institute (IAL), in August, 2017 [1]. Its first activity in 2018 was to record video lessons about “Research Integrity” (Integridade na Pesquisa Cientifica) in the Lato Sensu Specialization Course of IAL. The Center for Formation (CEFOR), a department of the Secretary of Health of São Paulo State, selected institutions to provide units of support for teaching specialization courses: IAL, Pasteur Institute, Health Institute and Butantan Institute (IB). The course “Laboratory Surveillance in Public Health”, designed by IAL, was conceived with theoretical and practical curricular components aimed at qualifying professionals to act in the Public Health laboratory. The program was divided into modules, with the “Ethics” discipline (including the sub-module “Integrity”) as part of the common theoretical module. The aims of this work were a) to describe the use of video lessons and the active teaching methodology approach to “Research Integrity (RI)”; b) to explore the advantages and disadvantages of these tools; c) to summarize evaluations about the use of these methodologies to promote RI. A Moodle platform was provided to allow students access both in the classroom and on their cell phones, tablets and notebooks to video lessons, additional supporting materials and the final test for the course. Video lessons, presented on the day of the sub-module “Integrity”, were also viewed by students who were in IB and regional units of IAL in twelve cities all over SP state. The classes had complementary activities: Group discussions on RI articles, debates about scientific misconduct cases and movie sessions to promote reflection and discussion on Ethics and Integrity. Our work team had a unanimous evaluation that the use of these methodologies and innovative resources brought new dynamics to our teaching process at IAL, allowing a better involvement of the students with themes of Research Integrity.

**Reference**

1. Ordinance establishing the Research Integrity Committee of Adolfo Lutz Institute. [Portaria de Criação do Comitê de Integridade na Pesquisa do IAL]. [Accessed 2018 Jun 6]; Available at: http://www.ial.sp.gov.br/resources/editorinplace/ial/2017_8_15/portaria_dg_ial_16_de_criacao_do_cipial_31_07_2017.pdf.

**Keywords:** Research integrity, teaching methodologies.

## 6 Research ethics committees at the Goiano Federal Institute: Experiences, strategies and prospects

### Adriane Gomes^1^, Daniela Custódio^2^, Lara Coelho^2^, Marina Marques^2^, Roberto Sanda^3^, Tânia Araújo^2^

#### ^1^Campus Iporá; ^2^Rectory; ^3^Campus Urutaí, Goiano Federal Institute of Education, Science and Technology (IF Goiano)

##### **Correspondence:** Adriane Gomes

Focusing on ethics and integrity in research, in order to contribute to the scientific, economic and social growth, the Research Ethics Committee (CEP) and the Ethics Committee for the Use of Animals (CEUA) of the Goiano Federal Institute of Education, Science and Technology (IF Goiano), are interdisciplinary collegiate and independent entities and act according to national and international regulations and initiatives. Created in 2010, the CEP is associated with the National Research Ethics Commission (CONEP), to defend the integrity and dignity of the research subjects/participants and to contribute to the development of research within ethical standards. The CEP is a multidisciplinary team composed of 22 members, and since joining Brazil Platform (2015) has evaluated 172 research projects. The CEUA was created in December, 2013, linked to the National Council for Control of Animal Experimentation (CONCEA), with the aim of presenting the principles of conduct to guarantee the care and the ethical handling of animals used for scientific or educational purposes. The CEUA is composed of 7 members, from different professional categories, with public employees and a representative member of the Society for the Prevention of Cruelty to Animals (SPCA), and up to now has evaluated 119 research projects. Aiming to educate and instruct practitioners of scientific research according to the ethical and integrity doctrines, the committees carry out educational activities for students, personnel, and professors, and follow-up visits of the projects, for assessing and monitoring ethical aspects of research in all campuses of IF Goiano. Thus, committees are certain that researchers’ experiences are an important educational space and professional training for students [1, 2]. Also, based on this institutional experience, we consider that good scientific practices should be included earlier in the curriculum, emphasizing scientific and social responsibility and ethical formation [3, 4].

**References**

1. Hyytinen H, Löfström E. Reactively, proactively, implicitly, explicitly? Academics’ teaching conceptions of research ethics and integrity. J Acad Ethics. 2017; 15 (1): 23- 41.

2. Pró-Reitoria de Pesquisa e Pós-graduação (PUCRS). Ética e integridade na pesquisa [Internet]. Available at: http://www.pucrs.br/pesquisa/etica-e-integridade-em-pesquisa/#etica-em-pesquisa. [Accessed 2018 Jun 10].

3. Steneck NH. Fostering integrity in research: Definitions, current knowledge, and future directions. Sci Eng Ethics. 2006; 12 (1): 53-74.

4. Vasconcelos S, Watanabe E. Proceedings of the IV Brazilian Meeting on Research Integrity (IV BRISPE). Res Integr Peer Rev. 2017; 2(1):1-12.

**Keywords:** Research ethics, research integrity, professional training.

## 7 Sample size in studies with biodiversity of parasites: Ethical implications and alternatives

### Moisés Gallas^1^, Eliane F Silveira^2^

#### ^1^School of Biosciences, Pontifical Catholic University of Rio Grande do Sul, Porto Alegre, RS, Brazil; ^2^ Natural Sciences Museum, Lutheran University of Brazil, Canoas, RS, Brazil

##### **Correspondence:** Moisés Gallas

In the studies about parasite biodiversity, a higher number of hosts are usually necropsied to obtain a representative parasite richness. Besides that, an adequate number of hosts is required to determine the ecological parameters of the infections or infestations, and for the statistical tests [1]. Considering these premises, the studies about parasite communities require a higher number of hosts, which directly raises ethical questions. The main goal of this study was to obtain information about the number of hosts which have been used in parasitological studies of wild animals’ parasites from Brazil, conducted through a research in the Scielo database. The results were organized considering the following information: year of publication, vertebrate host group, source of biological material, host sample size (n) and parasite richness (*S*), the number of parasite species found in each host. Among 192 reports found, 35 were performed with wild hosts. The studies were made between 1999 and 2018; the predominant vertebrate group studied was fish (71.43%), followed by birds (14.28%), mammals (8.57%), amphibians (2.86%) and reptiles (2.86%). The greatest number of hosts utilized was observed in two studies with birds (n= 265 and 237); however, a small number of parasites was detected (*S*= 2 and 3, respectively). The smallest number of hosts was found in studies with mammals (n= 3 and 5), with a parasite richness of 1 species in each study. We suggest that parasitologists should seek alternatives concerning the use of elevated number of hosts, trying to reduce host sacrifices [2]. Alternatives to these situations are, for example, the use of animals killed on highways, the donation of specimens by institutions such as zoos or research institutions, cooperation between laboratories and the use of as few animals as possible in research. All these possibilities are recommended by Russell and Burch’s 3Rs [3].

**References**

1. Jovani R, Tella JL. Parasite prevalence and sample size: Misconceptions and solutions. Trends Parasitol. 2006 May;22(5):214-8.

2. Gallas M, Silveira EF, Périco E. *Cylicospirura* (*Cylicospirura*) *felineus* (Chandler, 1925) Sandground, 1932 (Nematoda, Spirocercidae) in *Leopardus geoffroyi* d'Orbigny & Gervais, 1844 (Carnivora, Felidae): First report from the State of Rio Grande do Sul, Brazil. Neotrop Helminthol. 2014 Jul-Dec;8(2):349-55.

3. Rivera EAB. Ética na experimentação animal. Rev Patol Trop. 2001 Jan-Jun;30(1):9- 14.

**Keywords:** Bioethics, helminths, vertebrates, diversity, 3Rs.

## 8 Promoting scientific integrity from the beginning: The essential role of FICSAE graduate program

### Laudiceia Almeida, Aline Pacífico Rodrigues, Anna Carla Goldberg, Luiz Vicente Rizzo

#### Research and Education Institute (IIEP), Hospital Israelita Albert Einstein (HIAE), São Paulo, SP, Brazil

##### **Correspondence:** Anna Carla Goldberg

Scientific integrity is still at initial stages in Brazil. Thus, developing the processes within the frame of a graduate program to promote the necessary awareness is essential and aims to involve the next generations of Brazilian scientists. The Masters and Doctorate Graduate Program in Health Sciences, begun in July, 2014, with 166 students enrolled up to now and 64 M.Sc. and 14 Ph.D. degrees concluded, offers a syllabus based on ethics and scientific integrity, which, in addition to regular classes and scientific activities, creates an environment leading to scientific reasoning and critical analysis by the students. Procedures begin already during enrollment. The student will be included only if his or her project is approved in two instances: institutionally by means of the submission to an online platform called Research Project Management System or SGPP, where scientific, strategic, and financial feasibility is evaluated. In addition, the proposed study must be approved by the ethics committees for human-subject research or for animal care. Mandatory classes range from basic concepts in ethics and bioethics, role of review boards, and responsible animal care to good research practices, including authorship, plagiarism, conflict of interest, issues of “publish or perish”, selection bias and correct data analysis, evidence-based medicine, and advanced statistics, aiming to achieve the best methodological standards. Students are further required to discuss research misconduct, peer review, and issues of scientific integrity. Not least important, all theses and manuscripts are screened for plagiarism using Turnitin and Ithenticate software, and all research projects can be audited, at any time, by the institutional scientific integrity committee. Taken together, these practices, already well-established and linked to the graduate program, have been structured to create an environment fostering transparency and follow-up of research quality and scientific output with responsible and ethical conduct.

**Keywords:** Scientific integrity, transparency, ethics, graduate program**.**

## 9 Scientific integrity at Einstein: An integrated process

### Anna Carla Goldberg, Oscar Pavão dos Santos, Jacyr Pasternak, Luiz Vicente Rizzo

#### Research and Education Institute (IIEP), Hospital Israelita Albert Einstein (HIAE), São Paulo, SP, Brazil

##### **Correspondence:** Anna Carla Goldberg

Official procedures of scientific integrity at Einstein have been functioning since June 2017, after a whole year of tests and adjustments. Activities include processing complaints coming in from an external, confidential channel, active auditing of ongoing studies (all research projects are registered on a proprietary online follow-up system), courses ministered to institutional undergraduate (Medicine and Nursing) and graduate programs, training administered at medical boards with a focus on use of human subjects, conflicts of interest, authorship, collaboration, plagiarism, and other responsible conduct practices, and bimonthly tips published in the institutional newsletter. An internal committee of three members representing major areas involved in research (including the hospital and diagnostics) meets weekly to assess ongoing procedures. All papers and theses (both qualifying and final versions), including those under scrutiny are subject to plagiarism check. In one year of activities, the committee completed 15 audits, involving at least 24 researchers and 5 graduate students, and 30 staff nurses or MDs. Of the total, 11 audits showed issues varying from the more serious absence of relevant documents to late or missing reports and inadequate data storage (for example: excel sheets). In all cases, deliberate misconduct was ruled out, but researchers involved took effective steps toward solving the identified flaws in their personal or departmental procedures. Moreover, feedback was an important part of these initiatives. The overall conclusion is that the work of the committee should not only be maintained as a hallmark of ethical conduct in research but also as an important part of improving compliance and good practices in the research carried out by the institution.

**Keywords:** Scientific integrity, auditing, permanent committee, anonymous reporting channel.

## 10 Psychological assessment: Returning research results

### Manuela P Lima, Valéria Gonzatti, Daiane S de Oliveira, Daiana M Schütz, Marina B Yates, Tatiana Q Irigaray

#### Department of Psychology, Pontifical Catholic University of Rio Grande do Sul (PUCRS), Porto Alegre, Rio Grande do Sul, Brazil

##### **Correspondence:** Manuela P Lima

Surveys involving the application of instruments restricted to psychologists in psychological evaluation are increasingly common in the academic field. This study aims to relate the practice of psychological assessment to the research context, addressing the ethical aspects involved in this relationship. In this scenario, psychological assessment is found in the methodological section, and it is performed to respond to one or more objectives in order to promote knowledge about a specific theme [3,4]. The ethical precepts involved in the procedures and in the process of psychological assessment must be observed, and researchers should be aware that the psychological assessment in the research should be focused especially on the evaluation process and not only on its results [1,2]. It is known that it is the psychologist's duty to provide participants the research results, either in group or individual format, as one of the ways to benefit the individual in this process [3,4]. However, it is currently not the practice adopted by all researchers and, furthermore, there is no established model of returning results to the research participants. This presentation aims to address some factors underlying this problem.

**References**

1. Conselho Nacional de Saúde. Dispõe sobre pesquisa envolvendo seres humanos. Resolução N°466/2012. 2012. Brasil. https://conselho.saude.gov.br/resolucoes/2012/Reso466.pdf

2. Conselho Nacional de Saúde. Dispõe sobre a pesquisa em Ciências Humanas e Sociais. Resolução N°510/2016. 2016. Brasil. http://conselho.saude.gov.br/resolucoes/2016/Reso510.pdf

3. Conselho Federal de Psicologia. Código de Ética Profissional dos Psicólogos. Conselho Federal de Psicologia. 2005. https://site.cfp.org.br/wp-content/uploads/2012/07/codigo-de-etica-psicologia.pdf

4. Conselho Federal de Psicologia. Dispõe sobre a pesquisa em Psicologia. Resolução N°016/00. Revogada em 2012. http://www.fiocruz.br/biosseguranca/Bis/manuais/qualidade/Cfp16-00.pdf. https://site.cfp.org.br/wp-content/uploads/2014/07/Resolu%C3%A7%C3%A3o-CFP-n%C2%BA-010-12.pdf

**Keywords:** Psychological assessment, research, ethical aspects.

## 11 SciELO journals: Do they use software to detect plagiarism?

### Edilson Damasio^1^, Jacqueline Leta^2^

#### ^1^Library, Department of Mathematics, State University of Maringá (UEM), Maringá, PR, Brazil; ^2^Science Education Program, Institute of Medical Biochemistry Leopoldo de Meis (IBqM), Federal University of Rio de Janeiro (UFRJ), Rio de Janeiro, RJ, Brazil

##### **Correspondence:** Jacqueline Leta

The increasing number of cases of misconduct in science [1-3] has led to the development and the diffusion of a large number of computer software programs which are helping journal editors to detect bad behaviors, especially plagiarism. Considering the central role of SciELO editors in the regional publishing process, a survey was sent to 209 journal editors of SciELO Latin America collections [4] in order to investigate their views on some research integrity practices, including the usage of plagiarism detection software. The present abstract focuses on the results of 171 respondents: 82 Brazilian editors-in-chief and 89 from other Latin American countries. Regarding the use of plagiarism detection software, 44 (53.7%) Brazilians and 45 (50.6%) from other countries declare that their journals don’t use such software in the publishing process. Editors indicate that the main reasons for not using it are: no resources for acquiring it, lack of knowledge about such tools and lack of technical staff to use it. Among editors that declared that they use a plagiarism detection software (38 Brazilians and 44 from other countries), 42 use iThenticate, Turnitin, CrossCheck, eTblast or other free software. Another 20 editors indicated use of specific methodologies, including searching bibliographic databases and the Internet. This picture contrasts with a recent SciELO Brazil note that points to the need for using some detection as well as the elaboration of a guideline for authors about plagiarism systems [5]. In addition, it suggests that confidence in the editorial process for at least part of this set of journals may be at risk since they do not use any mechanism to detect or prevent plagiarism, one of the most frequent instances of misconduct in science today.

**References**

1. Lafollette MC. Stealing into print: Fraud, plagiarism, and misconduct in scientific publishing. Los Angeles: University of California Press; 1996: 293 p

2. Steen RG. Retractions in the scientific literature: is the incidence of research fraud increasing? J Med Ethics. 2011; Apr 37(4):249-53. doi: 10.1136/jme.2010.040923

3. Van Noorden R. Science publishing: The trouble with retractions. Nature. 2011; Oct 5; 478(7367):26-8. doi: 10.1038/478026a

4. SciELO. Scientific Electronic Library Online. [Internet]. 2018; Jun 4. Available from: http://www.scielo.org

5. SciELO Brazil. Critérios, política e procedimentos para a admissão e a permanência de periódicos científicos na Coleção SciELO Brasil. [Internet]. São Paulo, SP: SciELO; 2017 Oct; cited 2018 Jun 2. Available from: http://www.scielo.br/avaliacao/Criterios_SciELO_Brasil_versao_revisada_atualizada_outubro_20171206.pdf

**Keywords:** Plagiarism prevention, plagiarism detection software, editors, SciELO journals, Latin America

## 12 We know little about plagiarism in science education vis-à-vis cultural factors in the training of teachers: A perspective from a group of science teachers in Brazil

### Christiane C Santos^1,2^, Mariana D Ribeiro^1^, Sonia MR Vasconcelos^1^

#### ^1^Science Education Program, Institute of Medical Biochemistry Leopoldo de Meis (IBqM), Federal University of Rio de Janeiro (UFRJ); ^2^Pedro II School, Rio de Janeiro, Brazil

##### **Correspondence:** Christiane C Santos

In a recent study [1], we suggest that the relationship between pedagogical practices at school and plagiarism in science education have been little explored in Brazil. However, the literature shows a gap in such knowledge for many countries. When we look for publications on plagiarism in education, we find plenty of references. For example, a search for plagiarism AND education in Scopus (2008-2017) yielded 791 publications. The same is not observed for plagiarism AND “science education” – 19 results. Our search for plagiarism in three journals in the field, *Journal of Science Teacher Education, Science Education* and *Journal of Science Education and Technology* 2017, yielded one result for the first, two for the second, and ten for the third, respectively. Among possible explanations is that there have been only a few scholars in science education working on plagiarism as a research focus. This seems to be a reasonable hypothesis when we consider, for example, that in Proceedings of *New Perspectives in Science Education,* “plagiarism” appears only twice, with one mention in a section on publication ethics. However, as Grinnell et al. [3] suggest, it is timely to address research integrity, including plagiarism, early at school. Contributing to this perspective, we show results of a focus group with science teachers from one of the most traditional federal schools in Brazil. Most participants acknowledged that plagiarism received little if any attention in their science training at the university. Some revealed that advice was given only by their supervisors. We discuss these results vis-à-vis lack of formal plagiarism policies in the training of teachers at a major federal university from which a number of these participants graduated.

**References**

1. Santos CC, Santos PS, Sant’Ana MC, Masuda H, Barboza MB, Vasconcelos SMR. Going beyond academic integrity might broaden our understanding of plagiarism in science education: A perspective from a study in Brazil. An Acad Bras Ciênc. 2017 May; 89(1 Suppl.):757-771.

2. Conference Proceedings. New Perspectives in Science Education 6th Edition; 2017 Mar. 16-17; Florence, Italy. Florence: libreriauniversitaria.it; 2017

3. Grinnell F, Dalley S, Shepherd K, Reisch J. High school science fair and research integrity. PLoS ONE 2017 Mar; 12(3): e0174252

**Keywords:** Plagiarism; science education; pedagogical practices

## 13 An overview of plagiarism research, policies, projects and its applications through approaches in academic/research integrity conferences: A 2015-2017 perspective

### Karina de Albuquerque Rocha^1^, Sonia MR Vasconcelos^1^, Marisa Russo^2^

#### ^1^Science Education Program, Institute of Medical Biochemistry Leopoldo de Meis (IBqM), Federal University of Rio de Janeiro (UFRJ), Rio de Janeiro, RJ, Brazil; ^2^Department of Philosophy, Federal University of São Paulo (UNIFESP), São Paulo, Brazil

##### **Correspondence:** Karina de Albuquerque Rocha

Conferences addressing academic/research integrity have attracted a multidisciplinary community. This audience includes researchers, specialists and non-specialists, educators, policy makers and students from many countries. Topics presented at these conferences include institutional efforts to address plagiarism, and national and cultural challenges. These contributions have gained more visibility with the publication of Proceedings. But which approaches do they offer? What are the main concerns? Are there remarkable features in terms of approaches, fields and countries? These are among the questions addressed in this study. We have collected data from major conferences held in the 2015-2017 period – for some of them the first Proceedings were published in 2015. Only those with calls for abstracts and publication of Proceedings or Proceedings-like compendia were included [1-5]. For the material collected, only those with “plagiarism” in the title were considered. The total number of abstracts collected was 57. They have been categorized by approach, field and country. In terms of approach, about 40% of the abstracts focus on detection issues. The other 60% are distributed among other categories including research/perceptions, education, policy and teaching. We have identified that the major fraction of the 57 contributions looks at plagiarism from a higher-education perspective. A few on basic education are from Brazil, an exception in this panorama. Our results are preliminary, as the work is ongoing. We believe this type of analysis offers a useful perspective on the way this multidisciplinary community has approached plagiarism. Trends emerging from these contributions can provide a broader view of how initiatives and interest in this topic evolve.

**References**

1. Abstract book. 5th World Conference on Research Integrity, May 28 - 31, Amsterdam, The Netherlands, 144 pages, 2017. Available at: https://wcrif.org/documents/41-abstract-book-5th- wcri-2017/fie

2. Glendinning I, Foltýnek T, Rybička J, editors. *Plagiarism Across Europe and Beyond 2017: Conference Proceedings*, May 24-26, Brno, Czech Republic: Mendel University; 267 pages, 2017.

3. O’Brien et al. *Proceedings of the 4th World Conference on Research Integrity*. Res Integ Peer Rev. 1:9, doi:10.1186/s41073-016-0012-9, 56 pages, 2016. Available at: https://researchintegrityjournal.biomedcentral.com

4. *Plagiarism Across Europe and Beyond 2015: Conference Proceedings*, June 10–12, Brno, Czech Republic: Mendel University, 223 pages, 2015.

5. Vasconcelos S et al. *Proceedings from the IV Brazilian Meeting on Research Integrity, Science and Publication Ethics (IV BRISPE)*. Res Integ Peer Rev. Art.12. BioMed Central, 12 pages, 2017. doi: 10.1186/s41073-017-0035-x

**Keywords:** Plagiarism, conference proceedings, research integrity

## 14 Good scientific practices: Knowledge and behaviors of undergraduate life sciences students

### Natállia RA da Silva^1^, Maria RCG Novaes^2^, Dirce B Guilhem^1^

#### ^1^Graduate Program in Health Sciences, University of Brasilia, Brasilia, Federal District, Brazil; ^2^Foundation for Teaching and Research in Health Sciences and School for Advanced Studies in Health Sciences (FEPECS/ESCS), Brasilia, Federal District, Brazil

##### **Correspondence:** Dirce B Guilhem

**Background**

Scientific integrity is an essential requirement for science [1,2]. Universities share responsibility for dissemination of contents focused on building core values and moral responsibilities, as trainers of future scientists [1,3,4].

**Aims**

Analyze the profile of scientific initiation life sciences students, as well as to know their attitudes and practices related to the research process, including design, ethical review, conduct and publication.

**Methodology**

For an observational cross-sectional study with quantitative approach, data were collected by interviewing life-sciences undergraduate researchers, participants in a PIBIC project (CNPq) between 2013 and 2016. Data were collected using a structured questionnaire, via face-to-face and electronic interviews.

**Results**

138 students were interviewed, from six higher-education institutions of the Federal District, of a total of twelve life-sciences fields. About 74% of respondents were female and 26% male. Regarding the knowledge and adoption of practices and ethical principles for the development of research, most of them seemed to know the importance of ethical review processes in design, and publication of results. However, it was found that even with this familiarity with good scientific practices, students did not always behave in the most responsible way. Faced with a hypothetical case of complaint about scientific misconduct, 42.75% were neutral about this statement and 19.56% said they would not report the deviation. Also in relation to the training process of students, there was a predominance of formal education through courses and classes. Although 88% of respondents said they had already had content or discussions about the subject, only 25% said they were aware of the documents about ethics in research with humans and animals. Nevertheless, they recognized the importance of complementary areas such as research and research groups, for research ethics training.

**Conclusion**

Good practices in research must be included earlier in the curriculum, throughout the guidance and training [4].

**References**

1. Lins L, Carvalho FM. Scientific integrity in Brazil. J Bioeth Inq. 2014 Sep;11(3):283- 7.

2. Macfarlane B, Zhang J, Pun A. (2012): Academic integrity: A review of the literature. Stud High Educ. 2012 Aug 02; 39(2): 339-358.

3. Oran NT, Can HÖ, Şenol S, Hadımlı AP. Academic dishonesty among health science school students. Nurs Ethics. 2016 Dec;23(8):919-931.

4. Pádua GCC, Guilhem D. Scientific integrity and research in health in Brazil: A review of literature. Rev bioét. 2015 Apr; 23 (1):123-37. http://www.scielo.br/pdf/bioet/v23n1/en_1983-8034-bioet-23-1-0124.pdf.

**Keywords:** Scientific integrity, scientific misconduct, ethics, research, scientific and technical activities

## 15 Publication bias in Brazilian doctoral dissertations

### Renan MVR Almeida, Pedro O Vianna, Vinicios S Guilherme, Yuri AT Silva, José F Rodrigues, Marcus VP Carvalho

#### Graduate Program in Biomedical Engineering, Alberto Luiz Coimbra Institute for Graduate Studies and Research in Engineering (COPPE), Federal University of Rio de Janeiro, Rio de Janeiro, Brazil

##### **Correspondence:** Renan MVR Almeida

**Introduction**

It has been pointed out that the publication of papers with “positive” (favorable to the intervention) results is relatively easier compared to that for negative findings [1,2]; and that half of the double-blind randomized studies (RCTs) ever performed have never been published [3]. This problem (“drawer effect” or “publication bias”) imposes serious problems for the development of scientific knowledge.

**Objective** To evaluate the existence of publication bias in Brazilian doctoral dissertations.

**Materials and methods**

A total of 28 doctoral dissertations (theses) from the years 2012-2013 were randomly selected from the online Thesis and Dissertations Ministry of Education/CAPES database. Two major subjects were searched: RCTs and epidemiological studies containing the term “risk factor”. The publication of an article from the thesis was determined by the researcher’s CV (in the national “Lattes CV” database, available at http://buscatextual.cnpq.br/) and by an internet search using the title of the thesis and the author's name. The following information was obtained: *Thesis*: year of publication; main result (positive: main effect statistically significant, negative: main effect not statistically significant as defined by p values in the thesis summary). *Article*: year of publication (up to June/2018); main result (as above). Items were evaluated by two researchers, and, in case of doubt, a third one was called. When the pertinent information was unavailable, the thesis was discarded and a new random draw was performed for its replacement.

**Results**

Among the 28 theses analyzed (18 risk factors, 10 RCTs) 19 claimed positive results. Among these 19, 47% were published, and among the 9 that claimed negative results, 55% were published (p-value = 0.68, 95% CI: [0.40-1.80]) A stratification by the “risk factor” and “RCT” groups presented similar results.

**Conclusion**

It was not possible to identify a publication bias for the theses analyzed. The relatively low percentage (~ 50%) of published works is noteworthy.

**References**

1. Easterbrook PJ, Berlin JA, Gopalan R, Matthews DR. Publication bias in clinical research. Lancet. 1991; 337(8746):867-72

2. Sterne JAC, Egger M. Investigating and dealing with publication and other biases in meta-analysis. Brit Med J. 2001; 323(7304): 101-5.

3. AllTrials. The AllTrials campaign. Available http://www.alltrials.net/find-outmore/why-this-matters/the-alltrials-campaign; access July 2018

**Keywords:** Publication bias, doctoral dissertations

## 16 Ethical outcomes in research: Issues about safety indicators and safety performance measurement

### Felipe Lando, Éder Henriqson, Francisco S Rodrigues

#### Graduate Program in Business Administration, Pontifical Catholic University of Rio Grande do Sul (PUCRS), Porto Alegre, RS, Brazil

##### **Correspondence:** Felipe Lando

Issues concerning ethical research go beyond good methods or integrity in data gathering and analysis; they also concern research aims and results. Good research practices may not represent good research outcomes when results could be employed as bad social artifacts. Examples can be seen in the safety science domain, mainly when people try to develop ways to make systems and organizations get closer to “desirable” performance boundaries pushing safety to the unknown acceptable boundaries [1]. In this sense, a theoretical essay is proposed aiming for discussion on what can be a threat to ethical research even when hidden behind the good cover of safer systems and theories. Articles recently published in safety science journals raise doubts about the approaches taken by researchers when trying to develop safety indicators and models [2,3]. Their goals have been pointed towards defining systems’ status quo and developing acceptable limits where systems can go with acceptable risks. Therefore, Perrow’s 1989 theory [4] remains surrounding complex sociotechnical systems, which makes us believe that the closer a system gets to its acceptable risk barriers, the closer it gets to its next catastrophic accident.

**References**

1. Rasmussen J. Risk management in a dynamic society: a modelling problem. Saf Sci. 1997; 27:183-213.

2. Bergstrom J, van Winsen R, Henriqson E. On the rationale of resilience in the domain of safety: A literature review. Reliab Eng Syst Saf. 2015; 141:131-141.

3. Dekker S. The bureaucratization of safety. Saf Sci. 2014; 70: 348357.

4. Perrow C. Normal accidents: Living with high risk technologies. Second edition. Princeton: Princeton University Press; 1999. 464 p

**Keywords:** Safety science, ethical safety, safety indicators

## 17 Comparing quality of reporting between preprints and peer-reviewed articles in the biomedical literature

### Clarissa F D Carneiro^1^, Victor G S Queiroz^1^, Thiago C Moulin^1^, Olavo B Amaral^1^ and the Preprint Reporting Quality Consortium^2^

#### ^**1**^Institute of Medical Biochemistry Leopoldo de Meis, Federal University of Rio de Janeiro, Rio de Janeiro, RJ, Brazil; ^2^Carlos A.M. Carvalho, Seção de Arbovirologia e Febres Hemorrágicas, Instituto Evandro Chagas, Ananindeua, Pará, Brasil and Departamento de Morfologia e Ciências Fisiológicas, Universidade do Estado do Pará, Belém, Pará, Brasil and Faculdade Metropolitana da Amazônia, Belém, Pará, Brasil; Clarissa B. Haas, Department of Neuroscience, Section Medical Physiology, University of Groningen, University Medical Center Groningen (UMCG), Groningen, The Netherlands; Danielle Rayêe, Biomedical Sciences Institute, Federal University of Rio de Janeiro, Brazil; David E. Henshall, University of Edinburgh Medical School, Scotland, United Kingdom; Evandro A. De-Souza, Programa de Biologia Molecular e Biotecnologia, Institute of Medical Biochemistry Leopoldo de Meis, Federal University of Rio de Janeiro, Brazil; Felippe Espinelli, Institute of Medical Biochemistry Leopoldo de Meis, Federal University of Rio de Janeiro, Brazil; Flávia Z. Boos, PPG Psicobiologia, Universidade Federal de São Paulo, Brasil; Gerson D. Guercio, Biomedical Sciences Institute, Federal University of Rio de Janeiro, Brazil; Igor R. Costa, Institute of Medical Biochemistry Leopoldo de Meis, Federal University of Rio de Janeiro, Brazil; Karina L. Hadju, Laboratório de Biologia do Câncer, Biomedical Sciences Institute, Federal University of Rio de Janeiro, Brazil; Martin Modrák, Institute of Microbiology of the Czech Academy of Sciences; Pedro B. Tan, Institute of Biomedical Sciences, Federal University of Rio de Janeiro, Brazil; Steven J. Burgess, Carl R Woese Institute for Genomic Biology, University of Illinois at Urbana-Champaign, Urbana, Illinois, USA; Sylvia F.S. Guerra, Instituto Evandro Chagas, Secretaria de Vigilância em Saúde, Ministério da Saúde; Vanessa T. Bortoluzzi, Programa de Pós-graduacão em Ciências Biológicas: Bioquímica, Departamento de Bioquímica, Instituto de Ciências Básicas da Saúde, Universidade Federal do Rio Grande do Sul, Brasil

##### **Correspondence:** Clarissa F D Carneiro

Scientific communication is evolving rapidly with the rise of new technologies, but peer review remains mostly unquestioned. The use of preprint publishing has been the norm in some fields of science [1], and has recently risen in popularity among biomedical researchers [2]. Still, the quality of unreviewed articles raises doubts among some critics [3]. Research quality is a difficult feature to evaluate in scientific papers, mostly due to subjectivity. Reporting quality, however, can provide a more objective measure, as it can be summarized by a list of necessary information about an experiment that allows the reader to adequately appraise the results and reproduce them if needed. Peer review is usually not able to change experimental designs, but it could change their reporting, thus improving scientific communication. In this study, we compiled a list of measures based on previous studies assessing reporting [4-6] in order to provide a reporting quality score (range 0-100) for articles using cells, non-human animals or humans as the biological model, which were obtained randomly from PubMed or BioRxiv. A preliminary analysis showed no significant difference between groups (raw mean difference = 2.9, t-test p- value=0.6, n=10/group). Based on this result, we calculated a sample size of 76 articles/group to be able to observe a difference of at least 10% with 90% power. Evaluation of articles is being performed through a crowdsourced initiative, and each article will be evaluated online by 3 independent collaborators, allowing us to assess the most prevalent answer for each question. The complete methods have been preregistered at https://osf.io/tksmx/. Analyses are in progress and due for completion by August 2018. This study may provide empirical evidence on one of the most central paradigms in the scientific enterprise and guide future efforts to improve scientific communication.

**References**

1. Ginsparg P. (2011) It was 20 years ago today... Preprint at https://arxiv.org/abs/1108.2700v2.

2. Berg JM, Bhalla N, Bourne PE, Chalfie M, Drubin DG, Fraser JS, et al. Preprints for the Life Sciences. Science 2016; 1520:1–16. doi:10.1126/science.aaf9133

3. Calne R. Preprint servers: Vet reproducibility of biology preprints. Nature 2016; 535:493. https://www.nature.com/articles/535493b

4. Group TNC, Corresponding MRM. Findings of a retrospective, controlled cohort study of the impact of a change in Nature journals’ editorial policy for life sciences research on the completeness of reporting study design and execution. bioRxiv 2017; doi: 10.1101/187245

5. Kilkenny C, Browne WJ, Cuthill IC, Emerson M, Altman DG. Improving bioscience research reporting: the ARRIVE guidelines for reporting animal research. PLoS Biol. 2010; 8:e1000412. doi:10.1371/journal.pbio.1000412

6. Camarades G. A randomised controlled trial of an Intervention to Improve Compliance with the ARRIVE guidelines [Internet]. [cited 16 Oct 2017]. Available: https://ecrf1.clinicaltrials.ed.ac.uk/iicarus

**Keywords:** Preprints, peer-review, reporting quality

## 18 Scientific integrity: Issues addressed by Brazilian authors and publishers during the period of 2014-2107

### Gabriela C Cantisani^1^, Dirce B Guilhem^1,2^

#### ^1^Graduate Program in Health Sciences, University of Brasilia (UnB), Brasilia, Federal District, Brazil; ^2^Graduate Program in Nursing, College of Health Sciences, University of Brasilia (UnB), Brasilia, Federal District, Brazil

##### **Correspondence:** Dirce B Guilhem

**Abstract**

**Introduction:** Scientific Integrity assumes an important role in the development and dissemination of knowledge resulting of research studies [1,2,3]. On the other hand, scientific misconduct represents the adoption of questionable practices by researchers and academics and is increasing in recent years [3,4,5]. To guarantee reliability, transparency and best quality research it is important to include good ethical and scientific practices in the context of research

**Objective:** To analyze how the theme of scientific integrity has been addressed by Brazilian authors publications during period 2014-2017.

**Method:** Integrative literature review using PRISMA protocol [6]. Data collection occurred during December 2017 and January 2018 using three electronic databases: SciELO, LILACS/IBECS, and PubMed, including papers published from January 2014 to December 2017. The Editorials were included considering the relevant questions about scientific integrity addressed by them.

**Results and Discussion:** The analytical corpus consisted of 35 publications: 17 articles and 18 editorials. After the initial analysis, categories of scientific integrity emerged for discussion: 1) Scientific and/or academic fraud: 6 articles 2) Scientific Integrity concepts and/or outlook: 3 articles 3) Plagiarism: 4 articles, 4) Scientific Responsibility: 4 articles. The categories for editorial were 1) Scientific Misconduct: 7 editorials, 2) Plagiarism: 5 editorials, 3) Editorial Integrity: 4 editorials, 4) Authorship: 2 editorials.

Results reflects the evolution of the idea of how important it is the adoption of scientific integrity concepts during research as a guarantee of quality in science. The need for a standardized definition about the concepts is a consensus among the authors in Brazil.

**Conclusion:** The progress of discussions on scientific integrity in Brazil is moving forward, but publications still could be more embracing. Education, productive process of research, and insertion of policies of scientific integrity is among the most common issues discussed by authors. The production of empirical data on the national scenario is still incipient.

**References**

1. Pádua GCC, Guilhem D. Integridade científica e pesquisa em saúde no Brasil: revisão da literatura. Rev. Bioét. 2015 jan/abr [access: 11 janeiro 2018]; 23(1):124-38. Available at: http://www.scielo.br/pdf/bioet/v23n1/1983-8034-bioet-23-1-0124.pdf.

2. Godecharle S, Fieuws S, Nemery B. et al. Scientists still behaving badly? A survey within industry and universities. Sci Eng Ethics. Oct 2. [access: 31 Jan. 2018]. DOI: 10.1007/s11948- 017-9957-4.

3. Ferric C, Fang FC, Steen, RG, Casdecall A. Misconduct accounts for the majority of retracted scientific publications. PNAS 2012 [access: 31 Jan.2018]; 109(42)17028-17033. Available at: http://www.pnas.org/content/pnas/109/42/17028.full.pdf.

4. Chamon W, Dantas PEC. What is plagiarism after all? Editorial. Arq. Bras. Oftalmol. 2016 mar-abr. [access: 18 Jan. 2018]; 79(2):V-VI. Available at: http://www.scielo.br/scielo.php?script=sci_arttext&pid=S0004-27492016000200000&lang=pt. Acesso em 11 jan. 2018.

5. Moritz VR, Almeida KAR, Catelani F, Fontes-Pereira AJ, Vasconcelos SMR. Plagiarism allegations account for most retractions in major Latin American/Caribbean databases. Sci Eng Ethics (2016) 22:1447–1456.

6. PRISMA Statement. Transparent reporting of systematic reviews and meta-analyses. [Internet]. [access 22 Jan. 2018]. Available at: http://www.prisma-statement.org.

**Keywords:** Bioethics; integrative review; scientific Integrity; scientific misconduct; fraud

## 19 The adoption of good research practices at the Oswaldo Cruz Foundation and its role in research integrity

### Renata Almeida de Souza^1^, Saada Chequer-Fernandez^1,2^, Ivanete M Presot^1,3^

#### ^1^Oswaldo Cruz Foundation, Rio de Janeiro, RJ, Brazil; ^2^Oswaldo Cruz Institute, Rio de Janeiro, RJ, Brazil; ^3^René Rachou Institute, Belo Horizonte, MG, Brazil

##### **Correspondence:** Renata Almeida de Souza

The Oswaldo Cruz Foundation is a federal institution linked to the Brazilian Ministry of Health. Created in 1900, Fiocruz seeks to interconnect the fields of science, technology, and health, conducting activities that include the development of research and provision of laboratory reference services, as well as manufacturing of vaccines and drugs. Since there are no applicable international standards and/or regulations that guide the adoption of good practices in research, Fiocruz has adopted two documents: that published by the World Health Organization entitled Handbook: Quality Practices in Basic Biomedical Research [1] and that published by the São Paulo Research Foundation entitled Code of Good Scientific Practice [2]. These documents include questions on quality policy and staff responsibility, physical resources, prescriptive and descriptive documents, a calibration and maintenance program for equipment, research integrity among others. Initially, awareness-raising actions were carried out in the different Fiocruz units through seminars and workshops. We continue the dissemination through the formalization of the course titled: Good Research Practices (GRP). In this context, the requirement for integrity stands out, an important practice for the maintenance of the research, not only by the traceability but mainly by the actual evidence collected. The use of Registration Books (numbered and distributed in the institution) guarantees the originality and the important rescue of the raw data, methodology, and time line, as well as the names of the professionals involved. Important conduct of the collaborators is in the composition of the Prescriptive Documents that include the research project, Study Plans, and Standard Operational Procedures. In addition, the Descriptive Documents contain the results, records, and reports. For continuous improvement, the institution has invested in the annual self- assessment process, which is applied to all research laboratories, allowing the identification of opportunities for improvement and periodically outlining the plan to strengthen actions to implement Good Practices and Research Integrity.

**References**

1. World Health Organization. Handbook: Quality practices in basic biomedical research [Internet]. (Editione Editoração e Consultoria Científica S/C Ltda., Trad.). Belo Horizonte: CPqRR; 2010. Available from: www.cpqrr.fiocruz.br/temporario/qualidade/95700_Manual.WHO.pdf

2. São Paulo Research Foundation. Code of Good Scientific Practices. [Internet]. FAPESP, São Paulo, Brazil; 2014. Available from: http://www.fapesp.br/boaspraticas/FAPESP-Codigo_de_Boas_Praticas_Cientificas_2014.pdf.

**Keywords:** Quality, good research practices, research integrity

## 20 Investigating humanization competencies for educational management

### Ana M Hoernig, Breno A Hoernig Jr

#### Graduate Program in Education, La Salle University, Canoas, Rio Grande do Sul, Brazil

##### **Correspondence:** Ana M Hoernig

The present study has as its theme the competencies of humanization essential to educational management in the present time. The objective is to identify humanization competencies in contemporary educational management. The methodology used is of a qualitative nature with literature review pertinent to the theme to provide the theoretical background and discuss the results found. Bibliographical sources and articles come from searches on specialized websites, which allow the researcher to have direct contact with the subject matter. The results obtained suggest some relevant humanization competencies to compose the profile of the educational manager [1, 2]. The competencies that should be developed in the manager include the ability to diagnose and define problems, formulate objectives, generate solutions and establish activities necessary to achieve objectives, with autonomy, articulation, flexibility, respect for diversity, dialogue, dynamism and ethics [1, 3, 4, 6]. The search for humanization competencies in educational institutions, when they take place, generates a harmonious climate in the educational community [5]. Considering institutions as educational entities analogous to cells in the body, the units of education, such circumstances may trigger a positive effect that extends from communities to the whole of society [2].

**References**

1. Barros LM, Santos BS, Santos HJX. O desafio da formação do docente enquanto gestor educacional. Areté. Revista Digital del Doctorado en Educación de la Universidad Central de Venezuela. 2016; Jul-Dec. 2; 4; p. 25 - 40.

2. Brasil; Ministério de Educação/Secretaria de Articulação com os Sistemas de Ensino (MEC/SASE). Planejando a próxima década: Conhecendo as 20 metas do Plano Nacional de Educação, 2014, 63 p. Available at: http://pne.mec.gov.br/images/pdf/pne_conhecendo_20_metas.pdf

3. Libâneo JC. Organização e gestão da escola: Teoria e prática. 5th edition. Goiânia, GO, Brazil: Alternativa; 2004, 320 p.

4. Lück H. Concepções e processos democráticos de gestão educacional. Petrópolis, Brazil: Vozes, 9 ed. 2013, 136 p.

5. Nussbaum M. Sem fins lucrativos: Porque a democracia precisa das humanidades. São Paulo, SP, Brazil: WMF Martins Fontes; 2015, 154 p.

6. Farias, W. O líder integral: Porque o bom ser humano precede o bom líder. São Paulo, SP, Brazil: Integrare; 2014, 168 p.

**Keywords:** Manager profile, humanization competencies, educational management.

## 21 The Brazilian Reproducibility Initiative: A systematic assessment of Brazilian biomedical science

### Ana P Wasilewska-Sampaio, Clarissa FD Carneiro, Kleber Neves, Olavo B Amaral

#### Institute of Medical Biochemistry Leopoldo de Meis, Federal University of Rio de Janeiro, Rio de Janeiro, RJ, Brazil

##### **Correspondence:** Olavo B Amaral

The Brazilian Reproducibility Initiative is meant to provide a systematic assessment of the reproducibility of published findings in basic Brazilian biomedical science. Inspired by other initiatives, such as the Psychology Reproducibility Initiative [1] and the Reproducibility Project: Cancer Biology [2], our aim is to replicate between 50-100 experiments from published Brazilian articles, focusing on common methods and performing each experiment in multiple sites within a network of collaborating laboratories. The first step for our project is to generate a list of commonly used methods in Brazilian science. To do that, we searched the Web of Science database for articles in journals within areas fitting the life sciences spectrum. We then analyzed a random sample of 100 papers from 2017 and registered the experimental model and method/technique for outcome measurement in each experiment. Based on this initial screening, we selected ten methods for a large-scale, automated screening of life sciences articles from 1998 to 2017, filtering for papers in which the majority of authors, including the corresponding one, are affiliated with a Brazilian institution. The techniques selected at this point are cell viability assays (3-(4,5-dimethylthiazol-2-yl)-2,5-diphenyltetrazolium bromide [MTT] reduction), elevated plus maze, enzyme-linked immunosorbent assay (ELISA), flow cytometry, hemotoxylin-eosin (HE) staining microscopy, immunohistochemistry, open field exploration, reverse transcriptase-polymerase chain reaction (RT-PCR), thiobarbituric acid reactive substances (TBARS) and western blot. Experiments for inclusion will include both rodents and cell lines, which were the most common biological models in our sample.

The next step will be to randomly select articles that make use of each of the methods, using full-text screening tools, in order to build a representative sample of experiments to be analyzed in systematic reviews and evaluated as replication candidates. After selection, our plan is to reproduce each experiment in at least 3 laboratories, in order to estimate the replicability of Brazilian biomedical science.

**References**

1. Klein RA, Ratliff KA, Vianello M, Adams RB, Jr., Bahník Š, Bernstein MJ et al., 2014. Investigating variation in replicability: A “many labs” replication project. Soc Psychol 45: 142-152 Available at: http://dx.doi.org/10.1027/1864-9335/a000178

2. Errington T, Iorns E, Gunn W, Tan FE, Lomax J, Nosek BA 2014. An open investigation of the reproducibility of cancer biology research. eLife 3:e04333. Available at: http://dx.doi:10.7554/eLife.04333

**Keywords**: Brazilian Reproducibility, systematic reviews

## 22 Perceptions of graduate students in chemistry regarding the open-access movement and predatory journals

### Dayse CS Martins, Letícia M Costa, Maria H Araujo

#### Department of Chemistry, Universidade Federal de Minas Gerais, MG, Brazil

##### **Correspondence:** Dayse CS Martins

The dissemination of ideas and results of scientific research, through the publication of articles, is extremely important for the development of science. For this reason, the open access movement has gained strength in the last few years, since it allows the freely access to published scientific papers. However, the movement has become a target for unethical professionals that created journals to publish papers without proper review, and unfortunately the number of these journals, known as predatory, has grown steadily [1,2]. Surprisingly, for a number of reasons, the open access movement and predatory journals are not known by a great number of Masters and PhD students at the Chemistry Department of UFMG. This information has been obtained after a survey among forty- five new students of our graduate program. The open access movement was known to only 34% (15 students) of the students, of whom only 8 had a deep understanding of the subject and were able to identify a predatory journal. With these results we consider that these subjects should be touched in disciplines, seminars and courses within the university. We believe that only after this approach would students be able to make informed choices about where to send their work to be published and to identify reliable sources of information to be used in the development of their research project. The Graduate Program in Chemistry of UFMG, initiated a movement to discuss these and other topics related to integrity in scientific research, through the organization of events (symposium on ethics), lectures and the creation of disciplines on the subject.

**References**

1. Beall J. What I learned from predatory publishers. Biochem Med. 2017; 27(2):273-8.

2. Shen C, Björk B-C. ‘Predatory’ open access: A longitudinal study of article volumes and market characteristics BMC Med. 2015;13(230):1-15.

**Keywords:** Research, scholarly publishing, peer review, research integrity

## 23 Regulation of research in Education: The tensions between ethical autonomy and normative heteronomy

### Pedro Savi Neto^1^, Mónica de la Fare^1,2^

#### ^1^Graduate Program in Education; ^2^Graduate Program in Social Services, School of Humanities, Pontifical Catholic University of Rio Grande do Sul (PUCRS), Porto Alegre, RS, Brazil

##### **Correspondence:** Pedro Savi Neto

This work represents the third phase of an investigation carried out within a research group that investigates the relationship between education and research ethics. The research is centered on the analysis of the applicability of Resolution CNS 510/2016 [1], as well as its repercussions in the Humanities and Social Sciences (HSS). The methodology of the first phase of the research, of an exploratory nature, combined the documentary analysis with the opinion survey of leading researchers and vice-leaders of research groups in education of the South Region of Brazil through a web questionnaire, the second phase broadened the territorial scope of the research and, in this third phase, we are resuming the theoretical analysis of the empirical basis. The objective of this third phase is to present three sets of questions (philosophical, formative and normative) as elements to problematize the possibility of ethical autonomy in research as opposed to normative heteronomy, especially in the educational field, as part of the HSS. The philosophical question is related to the tension established between the need for freedom for the development of ethical behavior and the social pressure exerted on the individual, from the beginning of their formative course, to integrate them in a society dominated by the interests of capital [2]. Against this background, the intervention of law, which should in principle be subsidiary, functions as an artificial source of moral principles, with the consequence that the question is shifted to a field of dispute of unequal forces in areas that are subject to normalization, a fact that ends up favoring normative invasions of hegemonic spheres that are less significant politically [3]. This is the case of Resolution CNS 510/2016, a standard of a health agency that regulates the research in HSS, representing a normative interference.

**References**

1. Brasil. Resolução 510/2016: ética em pesquisa em Ciências Humanas e Sociais. [Resolution 510/2016: ethics in research in Humanities and Social Sciences]. Ministério da Saúde/Conselho Nacional de Saúde, Brasília, 2016.

2. Adorno TW. Educação e emancipação. [Education and Emancipation]. Rio de Janeiro, RJ: Paz e Terra; 1995, 192 p.

3. Diniz D. Ética na pesquisa em ciências humanas: Novos desafios. [Ethics in human sciences research: new challenges]. Ciênc Saúde Colet. 2008; 13: 417-426.

**Keywords:** Ethics, research/training, regulation, education

## 24 Long road to scientific integrity: A review of published retractions of health and life sciences research at Brazilian institutions

### Rafaelly Stavale^1^, Graziani I Ferreira^1^, Maria RCG Novaes^2^, Dirce Guilhem^1^

#### ^1^Department of Nursing, Faculty of Health Sciences, University of Brasilia, Federal District, Brazil; ^2^Department of Medicine, Advanced School of Health Sciences, Federal District, Brazil

##### **Correspondence:** Rafaelly Stavale

**Background**

Measures to ensure research integrity have been widely discussed due to its social, economic and scientific impact [1 - 4]. Most investigations on this subject focus on the role of authors and institutions to promote research transparency [3]; however, research integrity is reinforced by editorial policies for publication of articles or retraction notices when needed [5 - 6]. Hence, scientific journals play an important role to promote and ensure integrity [3].

**Objective**

This systematic review aimed to investigate the main reason for medical and life science research retractions of authors affiliated to Brazilian academic institutions. Quality, availability and accessibility to data regarding retracted papers from the publishers are described considering the *Committee on Publication Ethics* recommendations [5].

**Methods**

Two independent reviewers searched for retracted articles since 2004 in PubMed, Web of Science, BVS and Google Scholar databases. Indexed keywords from MeSH and DeCS in Portuguese, English or Spanish were used. Data was also collected from the Retraction Watch website (www.retractionwatch.com). This study was registered on the PROSPERO systematic review database (CRD42017071647).

**Results**

A final sample of 65 articles was retrieved; these were published in national (20) and international (35) journals. The types of documents found were erratum (1); retracted article (3); retracted article with a retraction notice (5); retraction notice with erratum (3); retraction notice (45). Assessment of the Retraction Watch website added 8 articles not identified by the search on the bibliographic databases. Among the retrieved articles, plagiarism was the main reason for retraction (60%). Missing data were found in 57% of the retraction notices. It was a limitation to this review.

**Conclusions**

The majority of the retraction notices did not conform to COPE’s recommendations. It is important to engage publishers and editors in research integrity discussions in order to promote transparency at all levels of research: from its idealization, planning, execution, and reporting, to possible retractions.

**References**

1. Decullier E, Huot GL, Samson G, Maisonneuve H. Visibility of retractions: A cross- sectional one-year study. BMC Res. Notes. 2013; [cited 2018 jan]; 6: 238. Available from: https://www.ncbi.nlm.nih.gov/pubmed/23782596

2. Van Noorden R. The trouble with retractions. Nature 2011; 478:26-28. doi:10.1038/478026a

3. Bar-Ilan J, Halevi G. Post retraction citations in context: A case study. Scientometrics. 2017; [cited 2018 apr]; 3(1): 547. https://doi.org/10.1007/s11192-017-2242-0

4. Neimark J. Life of attack. Christopher Korch is adding up the costs of contaminated cell lines. Science 2015; [cited 2017 nov] 347(issue 6225) 938-940. Available from: https://www.jillneimark.com/pdf/line-of-attack.pdf

5. Wager E, Barbour V, Yentis S, Kleinert S, on behalf of COPE Council. Retractions: Guidance from the Committee on Publication Ethics (COPE). Croat Med J. 2009; [cited 2018 mar]; 50(6):532-535. Available from: doi:10.3325/cmj.2009.50.532.

6. Breen KJ. Misconduct in medical research: Whose responsibility? Int Med J 2003;33(4):186-191.

**Keywords:** Research integrity, research misconduct, retractions**.**

